# Miro1 R272Q disrupts mitochondrial calcium handling and neurotransmitter uptake in dopaminergic neurons

**DOI:** 10.3389/fnmol.2022.966209

**Published:** 2022-12-02

**Authors:** Lisa Schwarz, Karan Sharma, Lorenzo D. Dodi, Lara-Sophie Rieder, Petra Fallier-Becker, Nicolas Casadei, Julia C. Fitzgerald

**Affiliations:** ^1^Department of Neurodegenerative Diseases, Hertie Institute for Clinical Brain Research, University of Tübingen, Tübingen, Germany; ^2^Institute of Pathology and Neuropathology, University of Tübingen, Tübingen, Germany; ^3^Institute of Medical Genetics and Applied Genomics, University of Tübingen, Tübingen, Germany; ^4^NGS Competence Center Tübingen, Tübingen, Germany

**Keywords:** Miro1, Parkinson’s disease, mitochondria, calcium, dopaminergic neuron

## Abstract

The Rho GTPase Miro1, located at the mitochondrial outer membrane is known to properly distribute mitochondria to synapses, aid calcium buffering and initiate PINK1-Parkin mediated mitophagy. Several heterozygous *RHOT1*/Miro1 variants were identified in sporadic Parkinson’s disease patients. Miro1 R272Q is located within a calcium binding domain, but the functional outcome of this point mutation and its contribution to the development of disease are unclear. To address this, we introduced a heterozygous *RHOT1*/Miro1 R272Q point mutation in healthy induced pluripotent stem cells. In dopaminergic neurons, Miro1 R272Q does not affect Miro1 protein levels, CCCP-induced mitophagy, nor mitochondrial movement yet causes the fragmentation of mitochondria with reduction of cristae and ATP5A. Inhibition of the mitochondrial calcium uniporter phenocopied Miro1 R272Q cytosolic calcium response to Thapsigargin in active neurons, a similar effect was observed during the calcium buffering phase in Miro1 knockdown neuroblastoma cells. Altered mitochondrial calcium regulation is associated with reduced mitochondrial respiration and reduced catecholamine neurotransmitter uptake. Synaptic changes are not coupled to dopamine distribution or dopamine transporters but are linked to Miro1 R272Q-related calcium handling *via* the mitochondria concomitant with defective dopamine regulation at the mitochondrial surface by monoamine oxidase. We conclude that the Miro1 R272Q heterozygous point mutation dampens mitochondrial-calcium regulation and mitochondrial capacity *via* events at the outer membrane that are sufficient to disrupt dopaminergic function.

## Introduction

Miro1 is a GTPase anchored in the outer mitochondrial membrane (Fransson et al., 2003) by a C-terminal transmembrane domain (Fransson et al., 2006). One of its main functions in mammalian cells is mediating mitochondrial transport along the cytoskeleton (Fransson et al., 2006; Russo et al., 2009; MacAskill et al., 2009a). In neurons, this is of special significance because mitochondria are mostly synthesized in the soma and hence need to be distributed to the cell’s periphery. Miro1 arrests mitochondria at sites of synaptic activity by calcium binding (Wang and Schwarz, 2009; Macaskill et al., 2009b). Since this is also essential in neuronal development (Nguyen et al., 2014; Lopez-Domenech et al., 2016), it is not surprising that no homozygous *RHOT1*/Miro1 loss of function mutations are reported in humans. This is supported by a study showing that Miro1 knockout is postnatally lethal in mice (Nguyen et al., 2014).

Although genome-wide association studies failed to identify *RHOT1* (the gene encoding Miro1) as a risk locus for Parkinson’s disease (PD) (Anvret et al., 2012; Saeed, 2018; Nalls et al., 2019), rare *RHOT1* variants identified by exome sequencing (Berenguer-Escuder et al., 2019; Grossmann et al., 2019) have provided insight into the complex biology of PD. One of the four heterozygous mutations found in sporadic PD patients (Berenguer-Escuder et al., 2019; Grossmann et al., 2019), Miro1 R272Q, lies within the ligand mimicking domain of the N-terminal EF-hand domain. The role of Miro1 in PD became more prominent after the finding that a subset of sporadic PD patient fibroblasts retains Miro1 at mitochondria upon mitochondrial depolarization (Hsieh et al., 2019). This is relevant, because Miro1 acts in the same signaling pathway as PD proteins PINK1, Parkin and LRRK2 (Weihofen et al., 2009; Wang et al., 2011; Sarraf et al., 2013; Hsieh et al., 2016; Klosowiak et al., 2016). Work is underway to test compounds that modulate Miro1 levels at the mitochondria (Hsieh et al., 2019) and we previously correlated levels of Miro1 to mitochondrial respiratory complexes in post mitotic neurons (Schwarz and Fitzgerald, 2022).

Prompted by studies showing the necessity of Miro1 or its EF-hand domain for mitochondrial morphology and calcium dynamics (Chang et al., 2011; Vaccaro et al., 2017; Nemani et al., 2018; Modi et al., 2019; Konig et al., 2021; Lopez-Domenech et al., 2021), we hypothesized that heterozygous Miro1 R272Q could impair mitochondrial quality *via* disruption of mitochondrial calcium homeostasis. We wanted to study whether disruption of calcium sensing in neurons could affect mitochondrial movement, and whether this could partly explain the neuronal dysfunction observed in the PD patients (Grossmann et al., 2019; Berenguer-Escuder et al., 2020). We previously introduced the heterozygous Miro1 R272Q mutation into healthy induced pluripotent stem cells (iPSCs) to generate an isogenic pair, characterized the cells and performed whole genome sequencing to show genomic integrity and genetic identity (Schwarz et al., 2021). In this study we found that mitochondrial transport is not affected by Miro1 R272Q, whereas mitochondrial energetics disrupted by a R272Q-calcium phenotype is concomitant with synaptic changes including disrupted neurotransmitter homeostasis.

## Materials and methods

### Generation of NPCs and differentiation in hDaNs

We used previously established and fully characterized Heterozygous Miro1 R272Q and isogenic control iPSCs (Schwarz et al., 2021). Small molecule-induced neural precursor cell (NPC) derivation from iPSC colonies and subsequent differentiation into human midbrain-specific neurons (hDaNs) was described in (Schwarz and Fitzgerald, 2022), adapted from Reinhardt et al. (2013). This protocol generates mid-brain specific dopaminergic neurons from iPSCs *via* NPC intermediates. Approximately 35–40% of the resulting MAP2 positive, mature neurons are tyrosine hydroxylase (TH) positive. hDaNs were used at day 16–19 for experiments. Treatments were diluted in maturation medium, untreated hDaNs received fresh maturation medium for the duration of the longest treatment. Mitophagy was induced with 10 μM CCCP for 2/4/6 (+ 10 μM MG132)/22 h.

### RT-qPCR

RNeasy kit (Qiagen, #74104) following the manufacturer’s instructions was used for RNA isolation and QuantiTect SYBR green kit (Qiagen, #204243) following the manufacturer’s instructions for RT-qPCR. Primer sequences are listed in Supplementary Methods Table 1.

### Immunofluorescence

hDaNs were stained following standard procedures with primary antibodies (MAP2 1:2000 (abcam, #ab5392), TH 1:1000 (Pel Freez #P40101-150), Tom20 1:200 (Santa Cruz, #sc17764)) and secondary antibodies (1:1000; Thermo Fisher, #A21449, #A11070, #A21463). For details and analysis of TH+ neurons see Supplementary Methods.

### Immunoblotting

hDaNs were lysed in either 1% Triton X-100 in PBS or in 50 mM Tris HCl pH7.5/150 mM NaCl/1 mM EDTA/0.5% TritonX-100, both supplemented with cOmplete protease inhibitor (Millipore Sigma, #11873580001) and PhosStop phosphatase inhibitor (Sigma-Aldrich, #4906837001), and homogenized by subsequent passes through needles (5x 20G/8x 25G/10x 27G or 5x 25G/10x 27G). Following standard procedures, proteins were separated and blotted onto PVDF membranes (Merck, #IPVH00010), then incubated with primary α-tubulin 1:5000 (Sigma, #AA13), α-vinculin 1:5000 (Sigma, #V9131), Miro1 1:500 (Thermo Fisher, #PA-42646), Tom20 1:1000 (Santa Cruz, #sc11415), Total OXPHOS 1:1000 (abcam, #ab110413), LC3 1:1000 (Novusbio, #NB100-2220), Mitofusin 1:1000 (Abcam, ab#57602), MAO-B 1:1000 (abcam, #ab137778) and secondary antibodies (1:10,000; Li-cor, #926–32,210, #926–32,213, # 926–68,071, # 926–68,070) and detected with an Odyssey CLx (Licor) using Image Studio software (Licor). Image Studio Lite Ver 5.2 (Licor) was used for quantification of intensity of bands.

### Analysis mitochondrial movement

Mitochondria labelled with MitoTracker were imaged under controlled environment (37°C/5% CO_2_) and analyzed using fiji (Rasband, W.S., ImageJ, U. S. National Institutes of Health, Bethesda, MD, USA). For details see Supplementary Methods.

### Mitochondrial morphology and membrane potential

Mitochondria labelled with MitoTracker green Image-iT™ TMRM were imaged under controlled environment (37°C/5% CO_2_). Mitochondrial morphology was assessed as previously described protocol (Merrill et al., 2017) with alterations. Mitochondrial membrane potential was calculated as the ratio of TMRM to MitoTracker green signal. For details see Supplementary Methods.

### Electron microscopy

hDANs were fixed in 2.5% glutaraldehyde in 0.1 M Sodium Cacodylate Buffer (pH 7.4, PLANO, Wetzlar, Germany), overnight at 4°C. For details see Supplementary Methods. Processed ultrathin sections of 60 nm were examined using an EM 10-Electron microscope (Zeiss, Germany). 80 EM images from three independent hDaN differentiations were numbered and mitochondria were counted blind. For details see Supplementary Methods.

### MitoTimer analysis

hDaNs transfected with pMitoTimer plasmid [Addgene, #52659; (Hernandez et al., 2013)] were imaged using Zeiss Imager.Z1 equipped with an ApoTome.2 and an AxioCam MRm. For details see Supplementary Methods.

### Flow cytometry

hDaNs were treated with 1 μM Staurosporine for 4 h, dissociated and stained with AnnexinV and 7-AAD (BioLegend, #640926) following the manufacturer’s instructions. Mean fluorescence was measured in phenol red-free maturation medium using a MACSQuant (Myltenyi Biotec) and corrected for background fluorescence.

### Respiratory analysis

Prior to measuring oxygen consumption rate (OCR) and extracellular acidification rate (ECAR) in a mitochondrial stress test using a Seahorse XF96 Extracellular Flux Analyzer (Agilent), hDaNs were treated with 20 μM Mitoxantrone (Arduino et al., 2017) for 2 h prior to the measurement. For details see Supplementary Methods.

### Calcium imaging

Cytosolic calcium was imaged using FLUO-4. Cells were pre-treated with the MCU inhibitor Mitoxantrone and calcium dynamics following inhibition of ER calcium stores was triggered using Thapsigargin. For details and image analysis see Supplementary Methods.

### Neurotransmitter transporter uptake assay

For neurotransmitter transporter uptake assay (Molecular Devices, #R8173, Jorgensen, Niescier et al., 2018), hDaNs at day 22 or 24 of differentiation were treated for either 24 h with 50 μM L-DOPA, 0.5 h with 5 μM ionomycin or 2 h with 20 μM Mitoxantrone in maturation medium. Uptake was measured following manufacturer’s instructions in Hank’s buffered salt solution for 45 min at 37°C with a 30 s interval using a SpectraMax M2e plate reader (Molecular devices) and normalized to t = 0.

### Dopamine staining and image analysis

hDaNs were stained with Dopamine (1:500, Immusmol, #IS1005), DAT (1:1000, Millipore, #MAB369) and MAP2 and Z-stacks were imaged using Zeiss Imager.Z1 equipped with an ApoTome.2 and an AxioCam MRm. For details see Supplementary Methods.

### Measurement of MAO enzyme activity

MAO enzyme activity was measured using a luminescence-based assay on isolated mitochondria and activity of citrate synthase was used as control. For details see Supplementary Methods.

### Transcriptomics

High quality RNA was isolated from independent hDaN differentiations as described above, subjected to PolyA enrichment followed by sequencing. For further procedure, see Supplementary Methods.

### Statistics

For statistical analyses, GraphPad Prism 9 (9.1.1) was used. Data are presented as mean ± standard deviation (SD) or SEM (Figure 3 only) and (log) normal distribution was tested using the Shapiro–Wilk test. Appropriate statistical tests were used as indicated in the figure legends.

## Results

### Miro1 R272Q has no effect on mitochondrial movement nor Miro1 degradation during mitophagy

**Figure 1:**
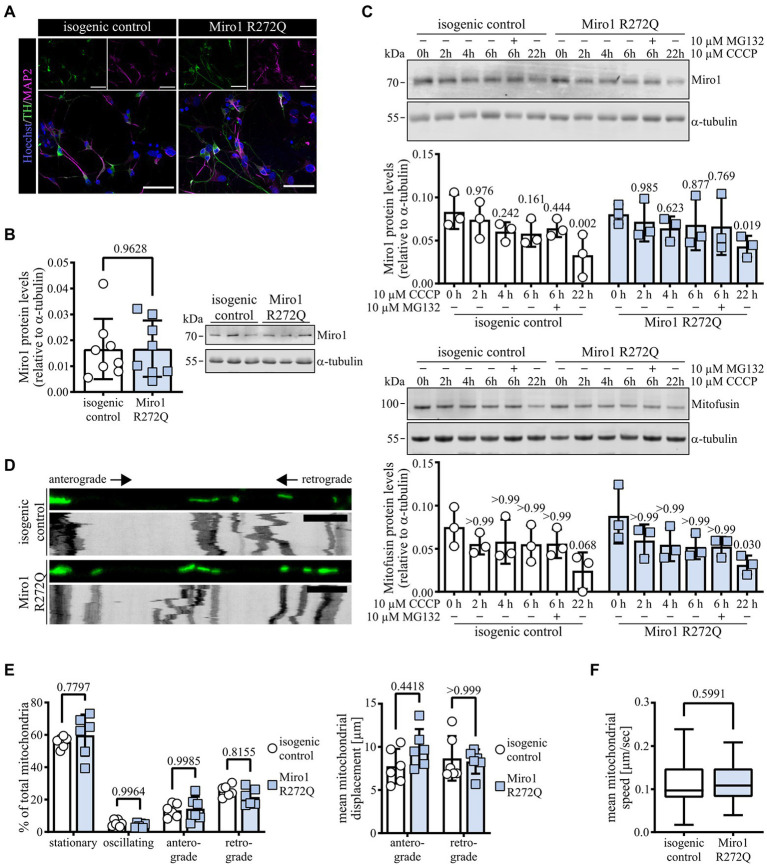
Miro1 R272Q does not change mitochondrial movement or Miro1 degradation upon CCCP-induced mitophagy. **(A)** Representative image of hDaNs stained with neuronal marker MAP2 and dopaminergic marker TH; n_Diff_ = 3. Scale bar: 50 μM. **(B)** Miro1 protein levels in hDaN lysates. Representative blot and quantification of intensity of Miro1 bands relative to a-tubulin. n_Diff_ = 8, data displayed as mean ± SD; paired t test (two-tailed). **(C)** Miro1 and Mitofusin protein levels in hDaNs upon induction of mitophagy using 10 μM CCCP for 0/2/4/6 (+ 10 μM MG132)/22 h. Representative blot and quantification of intensity of Miro1 and Mitofusin bands relative to a-tubulin. n_Diff_ = 3, data displayed as mean ± SD; Miro1: Two-way ANOVA with Tukey’s multiple comparisons; Mitofusin: Friedman test with Dunn’s multiple comparisons. **(D)** Representative first frame of movie and derived kymograph for movement analysis of hDaNs stained with 100 nM MitoTracker green. Scale bar: 10 μM. **(E)** Kymograph analysis of mitochondrial movement to classify stationary/oscillating/anterograde/retrograde fractions and mean displacement of mitochondria. n_Diff_ = 3 (2 datasets per differentiation), data displayed as mean ± SD; Fractions: Two-way ANOVA with Šídák’s multiple comparisons; Displacement: Friedman test with Dunn’s multiple comparisons. **(F)** Mean mitochondrial speed analyzed using TrackMate fiji plugin. n_Diff_ = 3 (n_Processes_ = 60), data displayed as mean ± SD; Mann–Whitney test.

We previously established Miro1 R272Q and isogenic control iPSCs (Schwarz et al., 2021) which we differentiated into dopaminergic neurons. They show positive immunofluorescence for neuronal marker MAP2 and dopaminergic marker TH (Figure 1A); approximately 35–40% of MAP2+ cells are TH+ (Supplementary Figure 1B), which is similar to previously reported numbers (Seibler et al., 2011; Hartfield et al., 2014; Bus et al., 2020). We further confirmed expression of hDaN markers using RT-qPCR (Supplementary Figure 1A). To assess whether the mutation alters protein stability and expression, we first tested protein levels of Miro1 and expression of *RHOT1* and *RHOT2*. Miro1 R272Q hDaNs show similar levels of Miro1 protein (Figure 1B) as well as *RHOT1* and *RHOT2* mRNA (Supplementary Figure 1C) compared to isogenic control hDaNs. Because of its role in mitophagy (Wang et al., 2011; Lopez-Domenech et al., 2021), we next assessed Miro1 degradation by inducing mitophagy using the ionophore CCCP. In Miro1 R272Q hDaNs, Miro1 is degraded in a similar fashion as in the isogenic control (Figure 1C). Assessing Mitofusin degradation (Figure 1C) as a proxy of removal of other outer mitochondrial membrane targets, as well as LC3 (Supplementary Figure 1D) for the induction of autophagy, we found no differences between genotypes. To test alterations in mitochondrial movement under basal conditions, we imaged mitochondria in neuronal processes labelled with MitoTracker green. Analysis of derived kymographs (Figure 1D) showed no effect in the fractions of stationary, oscillating and anterograde/retrograde moving mitochondria (Figure 1E), as was the distance mitochondria travelled (Figure 1E) and their mean speed (Figure 1F). In summary, the heterozygous Miro1 R272Q mutation is not sufficient to affect Miro1 steady state protein levels, its degradation or mitochondrial movement.

### Miro1 R272Q induces mitochondrial fragmentation and changes to cristae organization

**Figure 2:**
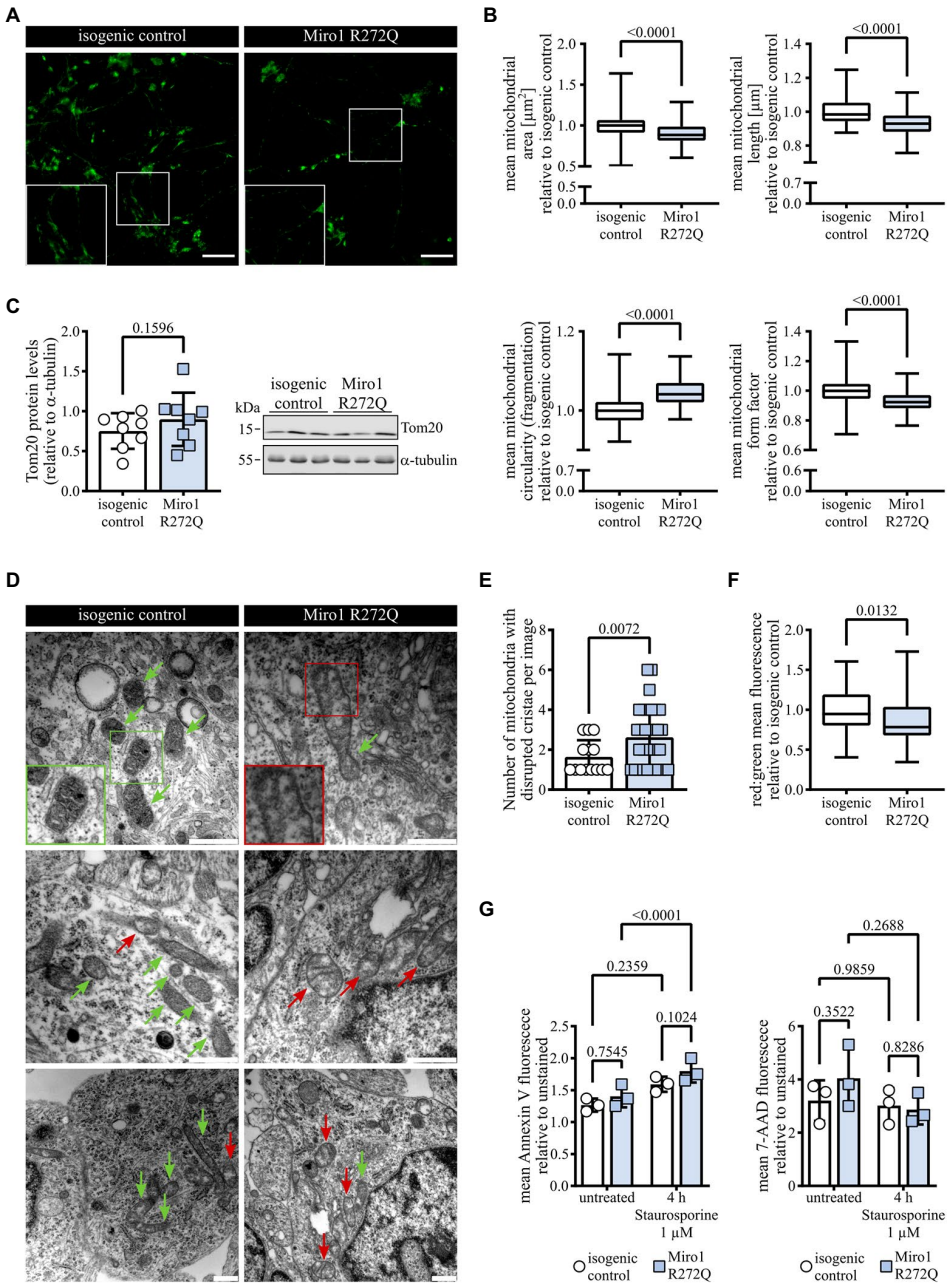
Miro1 R272Q alters mitochondrial morphology concomitant with changes in cristae organization. **(A)** Representative image of hDaNs stained with 100 nM MitoTracker green to label mitochondria for analysis of mitochondrial morphology. Scale bar: 50 μM. **(B)** Analysis of mitochondrial morphology of hDaNs stained with 100 nM MitoTracker green to assess mitochondrial area, length, fragmentation, and form factor. Values of one differentiation normalized to mean of isogenic control of the same differentiation. n_Diff_ = 3 (20 images per differentiation), data displayed as mean ± SD; Mann–Whitney test. **(C)** Tom20 protein levels in hDaN lysates. Representative blot and quantification of intensity of Tom20 bands relative to a-tubulin. n_Diff_ = 8, data displayed as mean ± SD; paired t test (two-tailed). **(D)** Representative electron microscopy of hDaNs. Red arrows indicate mitochondria with regions devoid of cristae, green arrows indicate mitochondria with maintained cristae. Scale bar: 500 nm. **(E)** Blinded quantification of mitochondria with disrupted cristae structure per EM image. n_Diff_ = 3, data displayed as mean ± SD; Welch’s t test (two-tailed). **(F)** Assessment of mitochondrial renewal in hDaNs by transfection with pMitoTimer. Quantification of mean red:green fluorescence and normalization of values of one differentiation to mean of isogenic control of the same differentiation. n_Diff_ = 3 (n_images_ = 42), data displayed as mean ± SD; Mann–Whitney test. **(G)** Flow cytometric assessment of susceptibility to apoptosis. hDaNs treated for 4 h with 1 μM Staurosporine were stained with apoptotic marker Annexin V and necrotic marker 7-AAD. Mean fluorescence of each channel normalized to unstained signal. n_Diff_ = 3, data displayed as mean ± SD; Two-way ANOVA with Tukey’s multiple comparisons.

Because R272Q lies in the first calcium-sensing domain and Miro1 calcium sensing was previously linked to mitochondrial morphology (Saotome et al., 2008; Nemani et al., 2018), we labelled mitochondria with MitoTracker green (Figure 2A) and analyzed mitochondrial morphology. Mitochondria in Miro1 R272Q hDaNs are significantly smaller and more fragmented compared to the isogenic control (Figure 2B). This did not result in a change of Tom20 protein levels (Figure 2C) which we assessed as a proxy of mitochondrial mass. Because Miro1 contributes to maintaining mitochondrial cristae structure (Modi et al., 2019), we assessed ultrastructure using electron microscopy. Compared to the isogenic control, mitochondria of Miro1 R272Q hDaNs had less cristae showing sections devoid of cristae altogether (Figure 2D). Blinded quantification of mitochondria with disrupted cristae shows that their number is significantly increased in Miro1 R272Q hDaNs (Figure 2E). Notably, the total amount of mitochondria was also increased (Supplementary Figure 1E). These findings indicate that morphological changes observed in Miro1 R272Q mitochondria might compensate for alterations in ultrastructure and do not affect the integrity of the mitochondrial outer membrane. To assess consequences of this phenotype, we next tested mitochondrial turnover using pMitoTimer (Hernandez et al., 2013) which relies on shifting from green to red fluorescence. In Miro1 R272Q hDaNs, the ratio between red and green is significantly decreased (Figure 2F) indicating an upregulation of mitochondrial turnover. Next, we treated hDaNs for 4 h with 1 μM Staurosporine to test whether Miro1 R272Q hDaNs are more susceptible to apoptosis. Staining with apoptotic marker Annexin V and necrotic marker 7-AAD revealed that Staurosporine significantly increases apoptosis in Miro1 R272Q, but not in isogenic control hDaNs without a concomitant increase in necrosis (Figure 2G). Taken together, Miro1 R272Q changes in mitochondrial ultrastructure trigger compensatory increase in mitochondrial turnover and heighten sensitivity to apoptosis. Although this might not be sufficient to cause PD, it burdens hDaNs and renders them more susceptible for other contributing factors for the pathogenesis of PD.

### Miro1 R272Q disrupts calcium handling into mitochondria and calcium buffering

**Figure 3:**
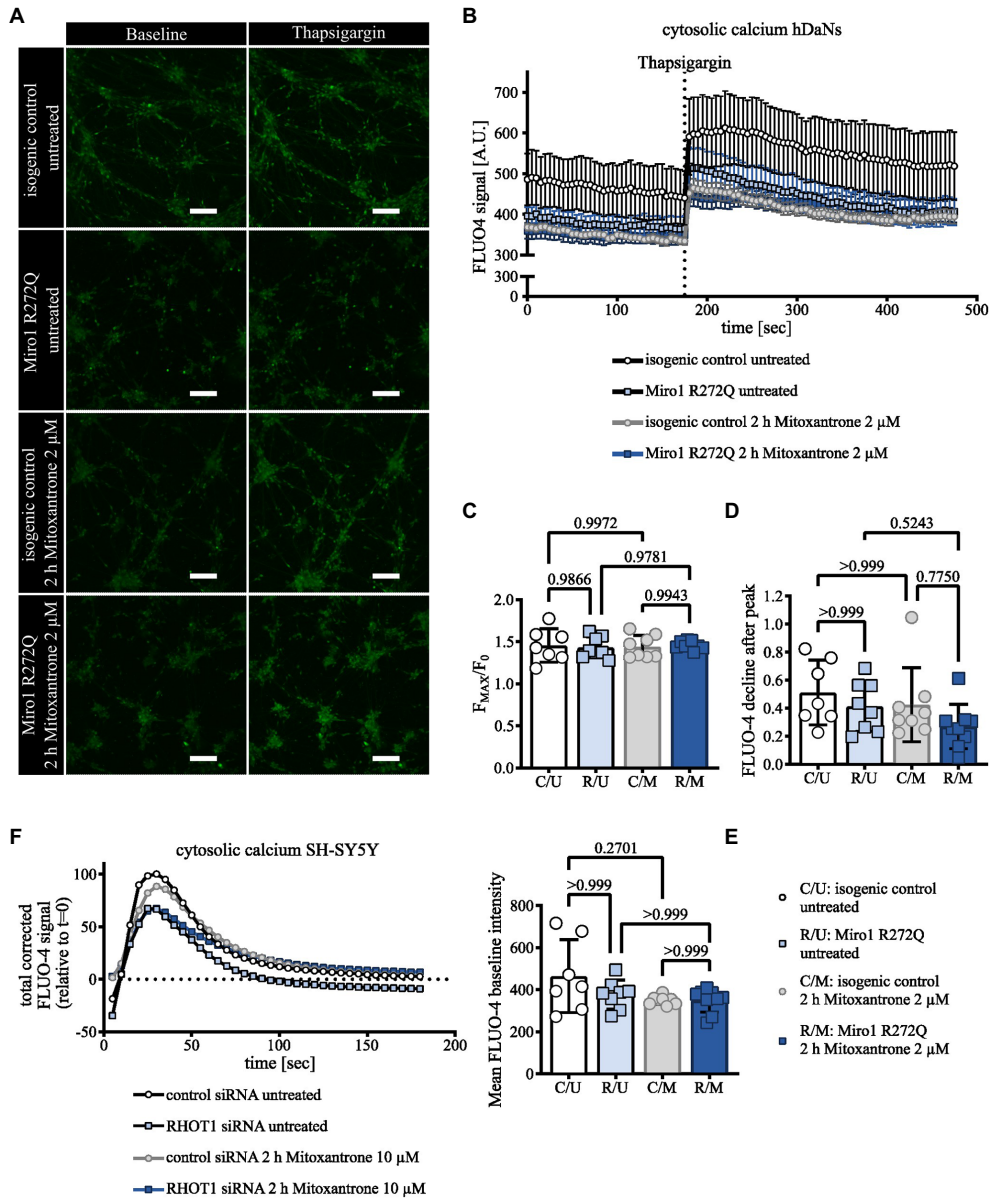
Miro1 R272Q alters calcium dynamics. **(A)** Representative Images of hDaNs before and after stimulation with 5 μM Thapsigargin. hDaNs were pretreated for 2 h with 2 μM Mitoxantrone and stained with cytosolic calcium indicator FLUO-4. **(B)** Cytosolic calcium trace in hDaNs before and after stimulation with 5 μM Thapsigargin. Quantification of total FLUO-4 signal. n = 7–9 (each n corresponds to 1 culture dish) obtained from 3 independent differentiations (n_Diff_ = 3), data displayed as mean ± SEM. **(C)** Quantification (from Figure 3B) of F_MAX_/F_0_, **(D)** reduction rate of FLUO-4 intensity after reaching peak intensity and **(E)** the mean baseline FLUO-4 signal. n = 7–9, n_Diff_ = 3, data displayed as mean ± SEM; One-way ANOVA with Tukey’s multiple comparisons for F_MAX_/F_0_; Kruskal Wallis with Dunn’s multiple comparisons for the rate of reduction of FLUO-4 signal and mean baseline FLUO-4 intensities. **(F)** Calcium trace in SH-SY5Y after stimulation with 5 μM Thapsigargin. SH-SY5Ys were transfected with non-targeting or RHOT1 siRNA to knockdown Miro1. SH-SY5Y cells were treated for 2 h with 10 μM Mitoxantrone. Cytosolic calcium was visualized using FLUO-4. Quantification of total corrected signal and normalized relative to t = 0. n = 2–3, data displayed as mean.

To assess whether changes in mitochondrial structure might be linked to altered calcium dynamics, we assessed calcium handling in hDaNs. We labeled cytosolic calcium using FLUO-4 and inhibited calcium uptake into the ER with 5 μM Thapsigargin under chelation of extracellular calcium. To link the cytosolic calcium response to mitochondrial calcium buffering, hDaNs were treated with 2 μM Mitoxantrone to specifically inhibit the mitochondrial calcium uniporter (Arduino et al., 2017). Miro1 R272Q hDaNs and hDaNs treated with the MCU inhibitor have on average less cytosolic calcium than the healthy control at baseline (Figures 3A,E). Inhibition of calcium uptake into the ER with Thapsigargin raises cytosolic calcium in hDaNs in all conditions (Figures 3A,B). Calculation of F_MAX_/F_0_ accounts for the maximal calcium levels after Thapsigargin treatment, suggesting that neither the R272Q genotype nor MCU inhibition significantly influences ER calcium uptake (Figure 3C). To minimize variability among neuronal populations, we measured only those neurons that were active (See Supplementary videos S1 and S2). Here we report a mild calcium phenotype caused by the Miro1 R272Q mutation. Following Thapsigargin treatment, Miro1 R272Q hDaNs immediately buffer calcium (Figure 3B) as opposed to an initial rise in cytosolic calcium observed in wild type cells (Figure 3B). MCU inhibition phenocopies the Miro1 R272Q mutation effect suggesting that even small changes to calcium dynamics in R272Q neurons could be attributed to mitochondrial calcium uptake. The rate of calcium buffering is not significantly affected by genotype nor treatment (Figure 3D). Co-culture of hDaNs with astrocytes could improve autonomous firing rates and reduce variability. Next we knocked down Miro1 in neuroblastoma cells and compared this to a non-targeting siRNA control transfection (Schwarz and Fitzgerald, 2022). Reduction of Miro1 protein levels in SH-SY5Y mildly decreases the cytosolic calcium peak in response to Thapsigargin and blocking the MCU with Mitoxantrone phenocopies the Miro1 knockdown in the mitochondrial buffering phase (Figure 3F). These data support previous findings that Miro1 modulates mitochondrial calcium uptake *via* the MCU (Chang et al., 2011; Niescier et al., 2018). The Miro1 R272Q mutation is located in the first EF-hand calcium binding domain of Miro1, we propose that this heterozygous mutation is sufficient to affect calcium handling involving the mitochondria. Development of methods to transduce iPSC-derived neurons or organoids with mitochondrial calcium indicators would help confirm this.

### Miro1 R272Q reduces mitochondrial respiratory capacity and neurotransmitter homeostasis

**Figure 4:**
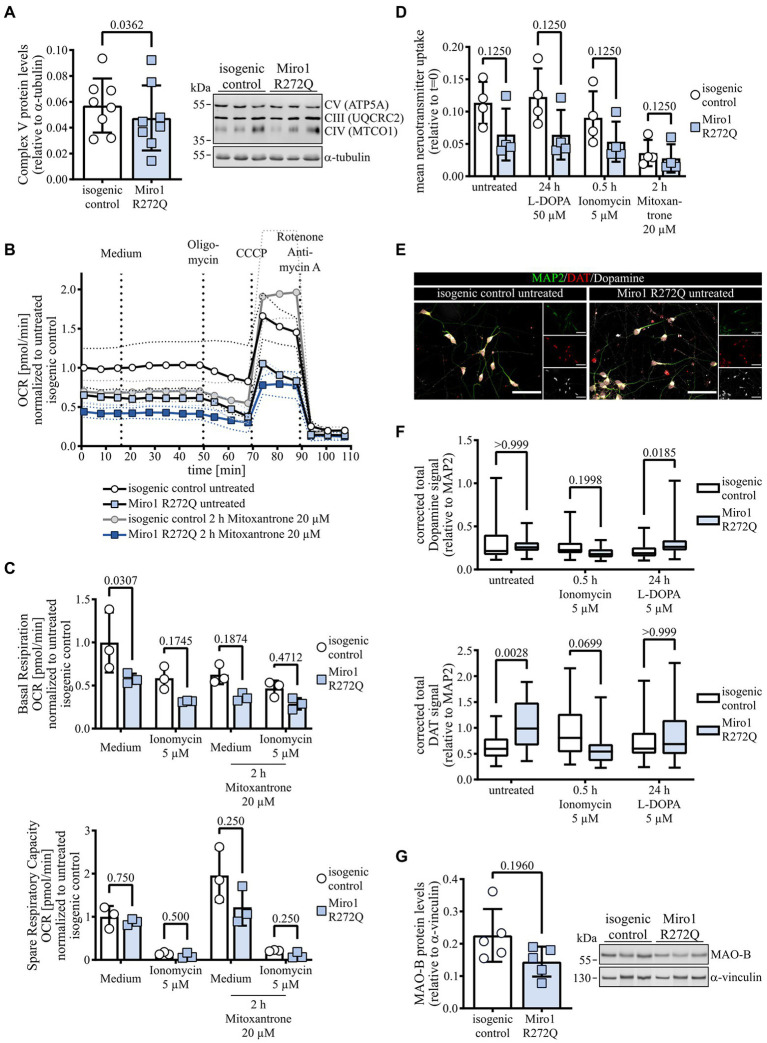
Impairment of mitochondrial calcium links to mitochondrial respiration and dopamine handling. **(A)** Complex V protein levels in hDaN lysates. Representative blot showing Complexes V, IV and III and quantification of intensity of Complex V bands relative to a-tubulin. n_Diff_ = 8, data displayed as mean ± SD; paired t test (two-tailed). **(B)** Respiratory analysis of hDaNs treated 2 h with 20 μM Mitoxantrone. Injection of medium, Oligomycin, CCCP and Rotenone with Antimycin A as indicated. Oxygen consumption rate was normalized to number of cells seeded and to mean of t = 0 in isogenic control. n_Diff_ = 3, data displayed as mean ± SD. **(C)** Basal respiration and spare respiratory capacity in response to injection of either medium or 5 μM ionomycin in hDaNs treated 2 h with 20 μM Mitoxantrone calculated from respiratory analysis. n_Diff_ = 3, data displayed as mean ± SD; basal respiration: Two-way ANOVA with Šídák’s multiple comparisons; spare respiratory capacity: Wilcoxon matched-pairs signed rank test with two-stage linear step-up procedure of Benjamini, Krieger and Yekutieli. **(D)** Neurotransmitter transporter uptake assay. hDaNs were treated either for 24 h with 50 μM L-DOPA, for 30 min with 5 μM Ionomycin or for 2 h with 20 μM Mitoxantrone. Slopes was calculated from uptake normalized to t = 0. n_Diff_ = 3, data displayed as mean ± SD; Wilcoxon matched-pairs signed rank test with two-stage linear step-up procedure of Benjamini, Krieger and Yekutieli. **(E)** Representative image of untreated hDaNs stained with Dopamine, DAT and MAP2. n_Diff_ = 3. Scale bar: 50 μM. **(F)** Quantification of corrected total Dopamine and DAT fluorescence normalized to MAP2. hDaNs were treated either for 30 min with 5 μM Ionomycin or for 24 h with 5 μM L-DOPA. n_Diff_ = 3 (n_images_ = 30), data displayed as mean ± SD; Kruskal-Wallis with Dunn’s multiple comparisons. **(G)** MAO-B protein levels in hDaN lysates. Representative blot and quantification of intensity of MAO-B bands relative to a-vinculin. n_Diff_ = 5, data displayed as mean ± SD; paired t test (two-tailed).

To test the functional consequences of both the altered mitochondrial structure and calcium handling observed in Miro1 R272Q hDaNs, we first assessed mitochondrial respiration. We tested levels of OXPHOS complexes and found a significant reduction of Complex V (Figure 4A), while Complexes IV and III were unaffected (Supplementary Figure 1F). Analyzing mitochondrial membrane potential using indicator TMRM, we found that the reduction in Complex V in Miro1 R272Q hDaNs does not affect mitochondrial membrane potential (Supplementary Figure 1G). We next measured mitochondrial respiration in a modified stress test; hDaNs were challenged with Oligomycin to inhibit Complex V, CCCP to depolarize mitochondria and Rotenone with Antimycin A to inhibit Complexes I and III, respectively. To link mitochondrial respiration to mitochondrial calcium, uptake was blocked with 20 μM Mitoxantrone for 2 h and prior to injection of Oligomycin hDaNs were first challenged with 5 μM Ionomycin to deplete mitochondrial calcium with medium as control. Miro1 R272Q hDaNs show an overall reduction of mitochondrial oxygen consumption (Figure 4B), but no compensatory increase in glycolysis indicated by extracellular acidification rate (Supplementary Figure 1H). Our data indicate that this is due to an impaired mitochondrial calcium uptake because depletion of mitochondrial calcium by either ionomycin or Mitoxantrone decreases respiration in isogenic control hDaNs to approximately 58% (Two-way ANOVA, Tukey’s multiple comparisons *p* = 0.0316) and 62% (Two-way ANOVA, Tukey’s multiple comparisons *p* = 0.0482), respectively (Figure 4C); basal respiration of Miro1 R272Q is reduced to approximately 58%. Miro1 R272Q does respond to both ionomycin and Mitoxantrone treatment, to approximately 32% (Two-way ANOVA, Tukey’s multiple comparisons *p* = 0.1687) and 37% (Two-way ANOVA, Tukey’s multiple comparisons *p* = 0.2818) of untreated isogenic control, respectively (Figure 4C), but could be due to the heterozygosity of the mutation. Assessing spare respiratory capacity (ability of mitochondria to meet energetic demand), we found no differences between Miro1 R272Q and isogenic control at baseline (Figure 4C). Treatment with Mitoxantrone however increases spare respiratory capacity in isogenic control hDaNs by a factor of approximately 2 (Friedman test with Dunn’s multiple comparisons *p* > 0.9999), while the increase in Miro1 R272Q is only approximately 1.4-fold (Friedman test with Dunn’s multiple comparisons p > 0.9999, Figure 4C). Taken together, these results support our hypothesis that Miro1 R272Q impairs mitochondrial calcium handling which in turn decreases mitochondrial respiration.

To better understand the effects of Miro1 R272Q on hDaNs, we performed transcriptomics (Supplementary Figure 2A). Using unbiased pathway analysis tools revealed neuronal pathways (Supplementary Figure 1 Table 1) which is highlighted by significant RNA expression changes in genes involved in synaptic and plasma membrane signaling (SYT2, DRD2, MAO-A, MAO-B) without significant changes to genes related to Miro1’s function related to mitochondrial transport *via* adaptors and kinesins, as well as mitochondrial calcium handling and quality control (Supplementary Figure 2B). Gene mapping using Qiagen Ingenuity software to visualize changes in specific pathways showed changes to gene expression in dopaminergic presynaptic signaling (Supplementary Figure 2C). We then tested dopamine handling in Miro1 R272Q hDaNs. Neurotransmitter uptake through monoaminergic transporters was decreased under baseline conditions in Miro1 R272Q to approximately 58% (Figure 4D). Treatment with dopamine precursor L-DOPA nor ionomycin significantly affects uptake in either of the hDaN lines (Figure 4D). However, inhibition of mitochondrial calcium uptake by Mitoxantrone inhibits neurotransmitter uptake further in Miro1 R272Q by approximately 34 percentage points (Friedman test with Dunn’s multiple comparisons p > 0.9999) hDaNs as well as the isogenic control to an approximately similar level of 31% (Friedman test with Dunn’s multiple comparisons *p* = 0.0682; Figure 4D). We next used immunofluorescence to assess the distribution of Dopamine and dopamine transporter (DAT) Miro1 R272Q and isogenic control hDaNs (Figure 4E). Overall dopamine levels are unchanged under baseline conditions in Miro1 R272Q, but we found a significant increase in amounts of the dopamine transporter DAT (Figure 4F). Ionomycin treatment does not alter dopamine levels but does reverse DAT levels in Miro1 R272Q (Figure 4F). Exposing hDaNs to dopamine precursor L-DOPA elevates dopamine levels in Miro1 R272Q (Figure 4F). Because previous work showed that monoamine oxidase (MAO)-A activity is sensitive to calcium concentration (Cao et al., 2009), we further assessed dopamine degradation by looking at MAO-A and MAO-B. RNA expression of both was significantly downregulated in Miro1 R272Q neurons (Supplementary Figure 3B). Titration of specific MAO-A and MAO-B inhibitors Clorgyline and Deprenyl, respectively, showed that hDaNs contain mostly MAO-B activity but also MAO-A (Supplementary Figure 3A), which is in line with the presence of both transcripts (Supplementary Figure 2B). In lysates of Miro1 R272Q hDaNs, this leads to reduced MAO-B protein (Figure 4G). Concomitant with this, enzyme activity and MAO-B protein levels in isolated mitochondria are reduced (Supplementary Figure 3B), which matches reduced enzyme at isolated mitochondria (Supplementary Figure 3C). We hypothesize, that the consequences of altered mitochondrial calcium uptake by Miro1 R272Q could affect calcium sensitive, dopamine regulating enzymes at the mitochondrial outer membrane in differentiated dopaminergic neurons. This combined with reduced mitochondrial respiratory capacity may be responsible for the synaptic changes identified by expression profiling.

## Discussion

Studies on *RHOT1*/Miro1 variants in sporadic PD patients (Berenguer-Escuder et al., 2019; Grossmann et al., 2019) underscored the relevance of Miro1’s role in PD although previous studies failed to provide a genetic link (Anvret et al., 2012; Saeed, 2018; Nalls et al., 2019). Miro1 acts in well studied biological pathways in PD, interacting with PINK1, Parkin, LRRK2 and α-synuclein (Wang et al., 2011; Birsa et al., 2014; Hsieh et al., 2016; Klosowiak et al., 2016; Shaltouki et al., 2018) and is an important marker of PINK1-Parkin mediated mitophagy and mitochondrial morphogenesis (Hsieh et al., 2019; Konig et al., 2021; Lopez-Domenech et al., 2021). We previously showed that the putative Miro1-PINK1 phosphorylation site Ser156 is important for regulating steady state levels of Miro1 and mitophagy flux (Schwarz and Fitzgerald, 2022) in differentiated cells. In this study the R272Q mutation did not influence Miro1 steady state levels and had no effect on Miro1 or Mitofusin degradation during six-hour treatment of hDaNs with CCCP. These findings are in contrast to Miro1 R272Q PD patient hDaNs, which showed reduced mitochondrial-lysosomal co-localization as a measure of mitophagy compared to healthy hDaNs (Berenguer-Escuder et al., 2019). We cannot rule out that the mutation influences Miro1 retention at the mitochondria with longer treatment or with Antimycin A or Oligomycin treatments that generate reactive oxygen species. It is also not clear whether something else, possibly the genetic background of the PD patient harboring the Miro1 R272Q variant could be driving additional biological burden. It will be interesting to see whether our observations can be replicated in gene corrected R272Q patient hDaN models and whether gene edited Miro1 R272Q animals display neurodegeneration, specifically of dopaminergic neurons in the *substantia nigra*. We hypothesized that the Miro1 R272Q mutation could be relevant in calcium-related Miro1 functions such as mitochondrial positioning because of its location within a calcium binding domain.

Work in primary hippocampal neurons proved that Miro1’s calcium sensing properties are necessary for mitochondrial positioning at synapses (Macaskill et al., 2009b) which drives presynaptic Ca^2+^ signals during homeostatic plasticity (Vaccaro et al., 2017). In this study, the Miro1 R272Q point mutation was found to have no influence on mitochondrial movement throughout neurites. Neuronal-specific Miro1 knockout in mice showed that Miro1 is not required for calcium-regulated mitochondrial movement but found reduced retrograde mitochondrial transport in axons and upper motor neuron development (Nguyen et al., 2014). Stephan and colleagues reported that Miro1 regulates intracellular calcium signaling through astrocyte-neuron interactions (Stephen et al., 2015). In this context it would be interesting to investigate heterozygous Miro1 PD variants in mouse or organoid models.

In contrast to work in Miro1 knockout mice (Nguyen et al., 2014), we found that the Miro1 R272Q point mutation reduced basal respiration of dopaminergic neurons and caused significant mitochondrial fragmentation. In line with evidence from Miro1 knockout studies in mice and mouse-derived neurons, our study reports that Miro1 R272Q does not impact critical mitochondrial functions such as mitochondrial membrane potential. Instead, the mitochondrial phenotype appears to compensate for reduced respiration tied to calcium deregulation at the mitochondrial outer membrane. Here we show that such mitochondrial compensation may involve mitochondrial fragmentation and reorganization of cristae. Previous studies showed lack of Miro1 EF-hand affects mitochondrial morphology (Saotome et al., 2008) and that Miro1 associates with the MICOS complex to maintain cristae structure (Modi et al., 2019). It is possible that Miro1 R272Q-induced depletion of ATP5A levels shown here trigger changes to the mitochondrial cristae since ATP synthase promotes curvature at cristae rims (Mukherjee et al., 2021). However, in electron microscopy images shown here, sections of unaffected lamellar cristae are still visible in addition to empty parts of the organelle, which would argue in favor of MICOS involvement. Interestingly, we did detect significantly reduced *CHCHD2* expression in Miro1 R272Q hDaNs. Mutations in *CHCHD2* are reported in PD in the Japanese population (Funayama et al., 2015) but other members of the CHCHD protein family are linked to regulation of cristae architecture. We concluded that Miro1 R272Q likely disrupts calcium sensing at the mitochondrial outer membrane, which will lead to reduced calcium stimulated respiratory flexibility. We suggest that this could induce further mitochondrial compensation *via* failure to properly engage the MCU. Calcium entry *via* the MCU preserves energy synthesis when the electron transport chain is impaired (Balderas et al., 2022).

We link disruption of Miro1 interaction with the MCU to regulation of mitochondrial calcium buffering. A previous study found that Miro1 interacts with MCU to modulate mitochondrial calcium uptake (Niescier et al., 2018) and we suggest that Miro1 is upstream of MCU. While MCU is regulated by MICU1 in the inner mitochondrial membrane by sensing matrix calcium (Mallilankaraman et al., 2012), we hypothesize that Miro1 senses cytosolic calcium to modulate uptake *via* the MCU, which has been shown to bind calcium with a very high affinity (Kirichok et al., 2004). This hypothesis is supported by our finding, that Miro1 R272Q does not affect mitochondrial membrane potential, which is a main driving force for MCU-mediated calcium uptake (Santo-Domingo and Demaurex, 2010). Miro1 R272Q dampens the capacity of mitochondria to buffer changes in cytosolic calcium affecting mitochondrial respiratory capacity as well as dopamine handling and synaptic signaling. A recent study tied mitochondrial calcium uptake to tuning of oxidative phosphorylation in neurons to meet the increase in energetic demand during synaptic activity (Ashrafi et al., 2020). Our data points to significant impact of the R272Q mutation on synaptic and dopaminergic function. We show less catecholamine neurotransmitter uptake in R272Q hDaNs despite more dopamine transporter. Since we observed normal distribution of dopaminergic markers and dopamine in both hDaN lines, we investigated whether dopamine degradation at the mitochondrial outer membrane could provide further evidence for the Miro1 R272Q calcium phenotype in PD. Significantly down-regulated MAO gene expression, protein levels and activity are in line with recent work suggesting that MAO activity stimulates mitochondrial respiration to support the bio-energetic demands of phasic dopamine release (Graves et al., 2020). Interestingly, calcium regulates the anchoring of MAOs at the mitochondrial surface, promoting the catalytic activity of MAO-A but not MAO-B (Ramsay and Albreht, 2021). In our study, both MAO-A and MAO-B are expressed in hDaNs, yet our inhibitor titration suggests that MAO-B enzyme activity is dominant. Further work is therefore needed to understand the stoichiometry of MAOs in Miro1 loss-of-function models and whether Miro1 interacts directly with MAOs at the mitochondrial outer membrane.

## Data availability statement

The original contributions presented in the study are included in the article/Supplementary material, further inquiries can be directed to the corresponding author.

## Ethics statement

The studies involving human participants were reviewed and approved by University of Tübingen. The patients/participants provided their written informed consent to participate in this study.

## Author contributions

JF: conceptualization, supervision, project administration, and funding acquisition. LS, KS, LD, L-SR, and NC: methodology. LD: software. LS, KS, LD, and L-SR: validation and investigation. LS, KS, LD, L-SR, JF, and NC: formal analysis. JF, PF-B, and NC: resources. LS, KS, LD, L-SR, PF-B, and NC: data curation. LS and JF: writing – original draft. LS, KS, and JF: writing – review and editing. LS: visualization. All authors contributed to the article and approved the submitted version.

## Funding

This study was funded by the Deutsche Forschungsgemeinschaft (DFG, German Research Foundation) Research Training Group MOMbrane 654651/GRK2364. NGS sequencing methods were performed with the support of the DFG-funded NGS Competence Center Tübingen (INST 37/1049–1).

## Conflict of interest

The authors declare that the research was conducted in the absence of any commercial or financial relationships that could be construed as a potential conflict of interest.

## Publisher’s note

All claims expressed in this article are solely those of the authors and do not necessarily represent those of their affiliated organizations, or those of the publisher, the editors and the reviewers. Any product that may be evaluated in this article, or claim that may be made by its manufacturer, is not guaranteed or endorsed by the publisher.
