# Supplementary material for: Miro1 R272Q disrupts mitochondrial calcium handling and neurotransmitter uptake in dopaminergic neurons
Source: Front Mol Neurosci. 2022 Dec 2;15:966209. doi: 10.3389/fnmol.2022.966209 (PMC9757607; doi:10.3389/fnmol.2022.966209)
Supplement: Supplementary file 1 [file Data_Sheet_1.docx]

Supplementary Figures


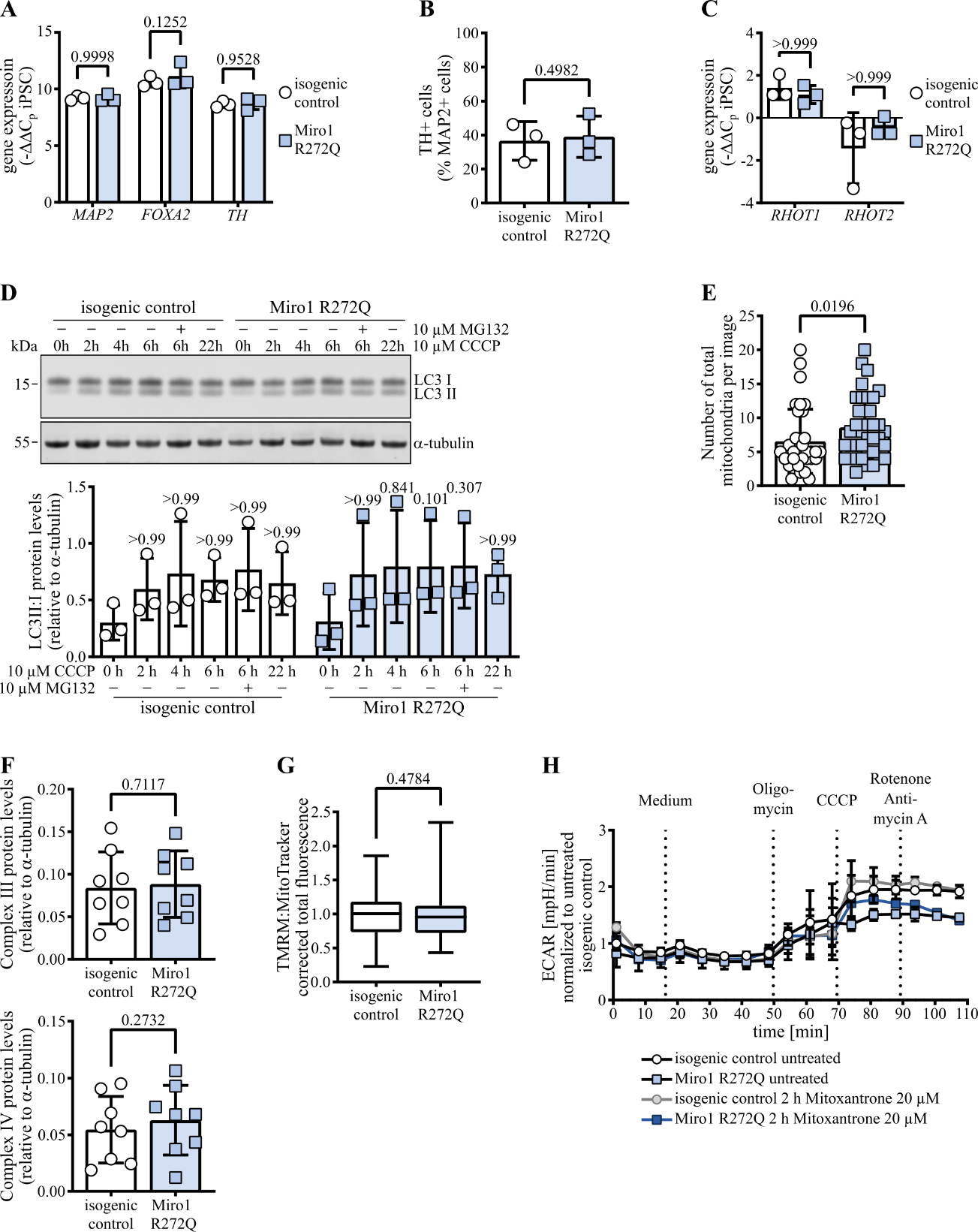


Supplementary Figure 1: (A) Analysis of gene expression of hDaN markers MAP2, FoxA2 and TH using RT-qPCR. Calculation of -ΔΔC_t_ relative to iPSCs. n_Diff_=3, data displayed as mean ± SD; Two-way ANOVA with Šídák’s multiple comparisons. (B) Quantification of MAP+ and TH+ cells to assess percentage of TH+ cells. n_Diff_=3, data displayed as mean ± SD; paired t test (two-tailed). (C) Analysis of gene expression of RHOT1 and RHOT2 using RT-qPCR. Calculation of -ΔΔC_t_ relative to iPSCs. n_Diff_=3, data displayed as mean ± SD; Friedman test with Dunn’s multiple comparisons. (D) Ratio of LC3II to LC3I protein levels in hDaNs upon induction of mitophagy using 10 µM CCCP for 0/2/4/6 (+ 10 µM MG132)/22h. Representative blot and quantification of intensity of LC3II/I bands relative to α-tubulin. n_Diff_=3, data displayed as mean ± SD; Friedman test with Dunn’s multiple comparisons. (E) Blinded quantification of mitochondria total mitochondria per EM image. nDiff=3, data displayed as mean ± SD; Mann-Whitney test. (F) Complex III/IV protein levels in hDaN lysates. Quantification of intensity of Complex III/IV bands relative to α-tubulin. n_Diff_=8, data displayed as mean ± SD; paired t test (two-tailed). (G) Image analysis of mitochondrial membrane potential. hDaNs stained with 25 nM TMRM and 100 nM MitoTracker green. Quantification of corrected total fluorescence of TMRM:MitoTracker green. Normalization of values of one differentiation to mean of isogenic control of the same differentiation. n_Diff_=3 (n_images_=60), data displayed as mean ± SD; Mann-Whitney test. (H) Extracellular acidification rate measured during respiratory analysis. Injection of medium, Oligomycin, CCCP and Rotenone with Antimycin A as indicated. Oxygen consumption rate was normalized to number of cells seeded and to mean of t=0 in isogenic control. n_Diff_=3, data displayed as mean ± SD.


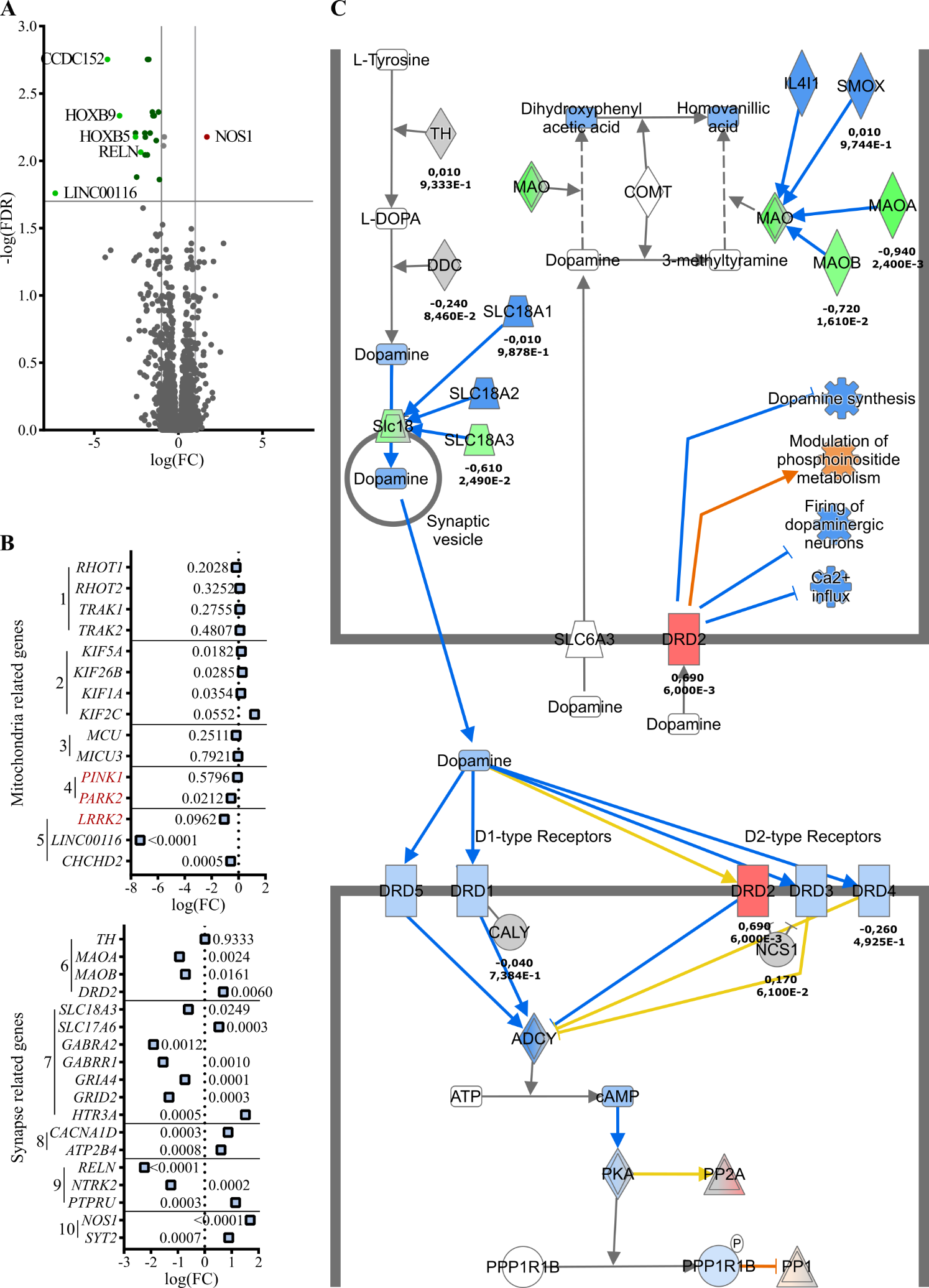


Supplementary Figure 2: (A) RNA sequencing of hDaNs. Volcano plot depicting differentially regulated genes in Miro1 R272Q compared to isogenic control. Plotted lines indicate threshold at -log(FDR) = 1.7 and log(FC) = ±1. Red dots indicate upregulation above threshold, green dots indicate downregulation below threshold. n_Diff_=3; data represents mean. (B) Expression of mitochondria and synapse related genes in Miro1 R272Q compared to isogenic control. Genes were grouped according to their function: (1) mitochondrial movement adaptors, (2) kinesins, (3) mitochondrial calcium uptake, (4) Mitochondrial quality control, (5) other mitochondrial functions, (6) dopaminergic neuron signaling, (7) other neurotransmitter-specific signaling, (8) neuronal calcium handling, (9) neuronal development, (10) other neuronal functions. Genes highlighted in red are PD-genes. Data shown as fold change to isogenic control. n_Diff_=3; data represents mean. (C) Ingenuity pathway analysis of differentially regulated genes in Miro1 R272Q compared to isogenic control. Pathways assessed were dopamine receptor signalling in the presynapse and synaptic long-term potentiation.

Supplementary Table 1: GOrilla pathway analysis of differentially regulated genes in Miro1 R272Q compared to isogenic control with p < 0.05 as target dataset and all genes detected in the RNA sequencing as background. List of top five results in categories function, process and component.

|  | **GO Term** | **Description** | **P-value** | **FDR** |
| --- | --- | --- | --- | --- |
| ***Function*** | [GO:0038023](http://www.godatabase.org/cgi-bin/amigo/go.cgi?query=GO:0038023&view=details) | signaling receptor activity | 9.05E-7 | 3.57E-3 |
|  | [GO:0060089](http://www.godatabase.org/cgi-bin/amigo/go.cgi?query=GO:0060089&view=details) | molecular transducer activity | 1.79E-6 | 3.53E-3 |
|  | [GO:0022839](http://www.godatabase.org/cgi-bin/amigo/go.cgi?query=GO:0022839&view=details) | ion gated channel activity | 1.84E-6 | 2.42E-3 |
|  | [GO:0022836](http://www.godatabase.org/cgi-bin/amigo/go.cgi?query=GO:0022836&view=details) | gated channel activity | 3.05E-6 | 3.01E-3 |
|  | [GO:0005509](http://www.godatabase.org/cgi-bin/amigo/go.cgi?query=GO:0005509&view=details) | calcium ion binding | 7.42E-6 | 5.85E-3 |
| ***Component*** | [GO:0031226](http://www.godatabase.org/cgi-bin/amigo/go.cgi?query=GO:0031226&view=details) | intrinsic component of plasma membrane | 2.14E-13 | 3.91E-10 |
|  | [GO:0044459](http://www.godatabase.org/cgi-bin/amigo/go.cgi?query=GO:0044459&view=details) | plasma membrane part | 1.54E-12 | 1.4E-9 |
|  | [GO:0005887](http://www.godatabase.org/cgi-bin/amigo/go.cgi?query=GO:0005887&view=details) | integral component of plasma membrane | 2.64E-12 | 1.61E-9 |
|  | [GO:0031224](http://www.godatabase.org/cgi-bin/amigo/go.cgi?query=GO:0031224&view=details) | intrinsic component of membrane | 4.6E-12 | 2.1E-9 |
|  | [GO:0044456](http://www.godatabase.org/cgi-bin/amigo/go.cgi?query=GO:0044456&view=details) | synapse part | 1.73E-11 | 6.31E-9 |
| ***Process*** | [GO:0023052](http://www.godatabase.org/cgi-bin/amigo/go.cgi?query=GO:0023052&view=details) | signaling | 1.75E-15 | 2.41E-11 |
|  | [GO:0009653](http://www.godatabase.org/cgi-bin/amigo/go.cgi?query=GO:0009653&view=details) | anatomical structure morphogenesis | 3.14E-15 | 2.16E-11 |
|  | [GO:0007267](http://www.godatabase.org/cgi-bin/amigo/go.cgi?query=GO:0007267&view=details) | cell-cell signaling | 7.43E-14 | 3.41E-10 |
|  | [GO:0032501](http://www.godatabase.org/cgi-bin/amigo/go.cgi?query=GO:0032501&view=details) | multicellular organismal process | 6.78E-13 | 2.33E-9 |
|  | [GO:0007155](javascript:toggle('elements_GO:0090183')?query=GO:0007155&view=details) | cell adhesion | 7.94E-13 | 2.19E-9 |


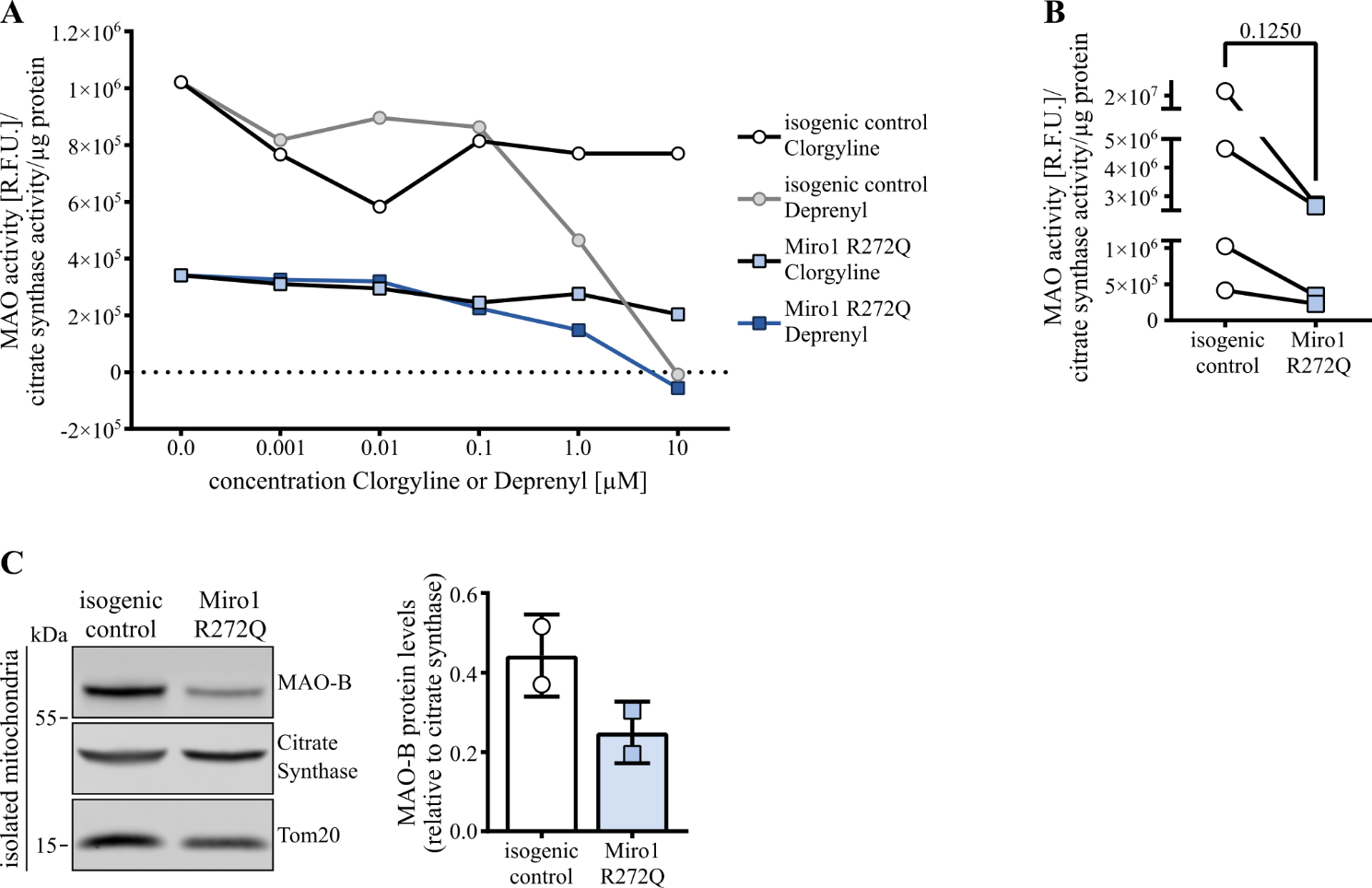
Supplementary Figure 3: (A) Analysis of MAO enzyme activity in mitochondria isolated from isogenic control and Miro1 R272Q hDaNs normalized to citrate synthase enzyme activity. MAO enzyme activity was measured in the presence of MAO-A and MAO-B inhibitors Clorgyline and Deprenyl, respectively, at the indicated concentrations. n_Diff_=1, data displayed as mean. (B) MAO enzyme activity in untreated mitochondria isolated from isogenic control and Miro1 R272Q hDaNs. n_Diff_=4, data displayed as mean ± SD. Wilcoxon test (two-tailed) (C) Representative blot showing MAO-B, citrate synthase and Tom20 in isolated mitochondria used for testing enzyme activity. Quantification of intensity of MAO-B band relative to citrate synthase. n_Diff_=2, data displayed as mean ± SD.
