# Supplementary material for: Miro1 R272Q disrupts mitochondrial calcium handling and neurotransmitter uptake in dopaminergic neurons
Source: Front Mol Neurosci. 2022 Dec 2;15:966209. doi: 10.3389/fnmol.2022.966209 (PMC9757607; doi:10.3389/fnmol.2022.966209)
Supplement: Supplementary file 2 [file Data_Sheet_2.docx]

Supplementary Methods

# RT-qPCR

Primer sequences are listed in Supplementary Table 1.

| Gene | Forward | Reverse |
| --- | --- | --- |
| GAPDH | CGAGATCCCTCCAAAATCAAG | GCAGAGATGATGACCCTTTTG |
| MAP2 | CCGTGTGGACCATGGGGCTG | GTCGTCGGGGTGATGCCACG |
| TH | TGTCTGAGGAGCCTGAGATTCG | GCTTGTCCTTGGCGTCACTG |
| MAP2 | CCGTGTGGACCATGGGGCTG | GTCGTCGGGGTGATGCCACG |
| FOXA2 | CCATGCACTCGGCTTCCAG | TGTTGCTCACGGAGGAGT |
| RHOT1 | TGTCACCCCAGAGAGAGTTC | GCCTGCTGTCTTTGTCTGTT |
| RHOT2 | ATTGAGACCTGCGTGGAGTG | AAGCGTTGAGCTCTTCGTCA |

# Immunofluorescence

hDaNs on Matrigel-coated coverslips were fixed in 4% PFA. For permeabilization for Tom20 staining, hDaNs were incubated 5 min with 0.5% TritonX-100 in PBS at room temperature. After blocking with 10% normal goat serum in wash buffer (0.01% TritonX-100 in PBS), antibodies were diluted in 5% normal goat serum in wash buffer and incubated over-night at 4°C (primary) and 1-2 h at room temperature. Hoechst (Molecular Devices, #H3569) was used to stain nuclei prior to mounting with Dako fluorescent mounting medium (Aligent, #S3023). Z-stacks were taken using a Zeiss Imager.Z1 equipped with an ApoTome.2 and an AxioCam MRm. Representative images of TH/MAP2 staining for characterization were projected with maximum intensity using ZEN software (blue edition, Zeiss) with subsequent adjustment of brightness and contrast. For analysis of TH+ neurons in fiji, z-stacks were projected with maximum intensity and brightness and contrast were adjusted (comparable between images). Counting of MAP2+ and TH+ cells was done using cell counter plugin. 10 images were counted per differentiation, (n_Diff_=3) and sum of MAP2+ and TH+ per differentiation was used to calculate percentage TH+ of MAP2+ cells; the percentage of one differentiation was used as a data point for statistics.

# Analysis of mitochondrial movement

Two to four days prior to imaging, 100,000 hDaNs were seeded on Poly-L-Ornithine (PLO, Sigma-Aldrich, #P8638-25MG)-Matrigel double-coated ibidi dishes. For labeling of mitochondria, hDaNs were stained for 7 min at 37°C with 5% CO_2_ with 100 nM MitoTracker green (Thermo Fisher Scientific, #M7514). For imaging, medium was replaced with phenol red-free maturation medium and a Leica DMi8 Microscope (40× objective) with the LASX software was used to acquire 61 frames (5 min/5 sec interval). Prior to selection of neurites for analysis, first and last frames were compared to exclude mobile neurites. After cropping, neurites were straightened (Kocsis et al., 1991) with maintenance of movement directionality (anterograde: left to right). For generation of kymographs, time lapses were re-sliced with default settings and then projected with maximum intensity followed by LUT inversion. For classification using displacement into stationary (≤ 2 µm), mobile (> 2 µm), or oscillating (≤ 2 µm and amplitude > 1.5 µm), the Pythagorean theorem was used for calculation. Per differentiation, two data sets were acquired. Per dataset representing one datapoint in the statistical analysis, the sum of all mitochondria analyzed per genotype was used to calculate fractions. Mean mitochondrial speed was measured using fiji-plugin TrackMate (Tinevez et al., 2017) after correcting bleaching with histogram matching and enhancing contrast by 2.5% in all slices. Calibration information was used for TrackMate analysis with LoG detector with an estimated blob diameter of 1 µm with sub-pixel localization and without threshold. Using HyperStack Displayer, automated filtering of spots was applied with manual adjustment where necessary. Particles were tracked using Linear motion LAP tracker searching a radius of 5 µm with a maximum frame gap of 2. Per dataset, 10 timelapses were analyzed, each of which was used as datapoint for statistical analysis.

# Mitochondrial morphology and membrane potential

Two to three days prior to imaging, 150,000 hDaNs were seeded on Matrigel-coated ibidi dishes. For labeling of mitochondria and indicating mitochondrial membrane potenital, hDaNs were stained for 7 min at 37°C with 5% CO_2_ with 100 nM MitoTracker green (Thermo Fisher Scientific, #M7514) and 25 nM Image-iT™ TMRM reagent (Thermo Fisher Scientific, #I34361), respectively. For imaging, medium was replaced with phenol red-free maturation medium and a Leica DMi8 Microscope (40× objective) with the LASX software was used to acquire Z-stacks with a slice interval of 0.88 µm. For analysis in Fiji, stacks of each channel were projected with average intensity and converted to 8-bit greyscale. Prior to generating binary images for the assessing mitochondrial morphology using and adapted protocol (Merrill, Flippo et al. 2017), images were convolved (0 0 −1 0 0/0 −1 −1 −1 0/-1 −1 24 −1 −1/0 −1 −1 −1 0/0 0 −1 0 0) with subsequent subtraction of background using a rolling ball radius of 10 px and unfast enhancement of local contrast with a blocksize of 9 and maximum slope of 4. Next, images were processed with the tubeness tool using sigma = 0.24156 with calibration information followed by despeckling, conversion into 8-bit and thresholding to create a binary image. Measuring area, shape descriptors, perimeter (used to calculate $\text{form factor=}{\text{perimeter}^{\text{2}}}/\text{4π ∙area}$) and fit ellipse of particles bigger than 1.5 px with a circularity of 0-1 was used to calculate the mean of all particles per image for each descriptor which was used as data point in statistics. TMRM corrected total fluorescence (CTF=IndDen_Particle_ - (area_Particle_ ∙ mean grey value_Background_) of each particle measured (bigger than 1.5 px and circularity 0-1) was normalized to MitoTracker green CTF. MitoTracker green binary images, generated by thresholding after subtracting background with a rolling ball radius of 15 px, were used to define the area for measuring area, integrated density and mean grey value of each channel. One datapoint in statistical analysis represents the mean TMRM:MitoTracker green CTF per image.

# Electron microscopy

Following fixation, samples were washed in 0.1 M Sodium Cacodylate Buffer and further fixed with osmium tetroxide (Sigma Aldrich). Samples were dehydrated by passing through a series of washes with increasing ethanol concentration (50%, 70%, 80%, 90%, 100% Ethanol, 30 min for each concentration). Samples were incubated in propylene oxide/Araldite overnight and then embedded by infiltration of pure Araldite. Semi-thin sections of 350nm were cut first using a Ultracut R-Microtome (Leica Microsystems, Germany) and stained with Richardson-Solution to identify regions of interest. Afterwards, the ultra-thin sections of 60nm were cut for electron microscopy.

# MitoTimer analysis

For transfection with pMitoTimer plasmid (Addgene, #52659; (Hernandez et al., 2013)) using Fugene HD (Promega, #E2311), according to the manufacturer’s instructions, 80,000 hDaNs per Matrigel-coated glass coverslip were seeded. Two days after transfection cells were fixed with subsequent staining with Tom20 as described above. For acquisition of 14 Z-stacks per differentiation with a slice interval of 0.72 µm and the same exposure times for each channel, a Zeiss Imager.Z1 equipped with an ApoTome.2 and an AxioCam MRm was used. In Fiji, stacks were projected with average intensity followed by conversion to 8-bit greyscale. Binary Tom20 generated by thresholding was used to define the area for measuring mean grey value of particles bigger than 1.5 px with circularity 0-1 of green and red channels. The mean of each channel per image was used to calculate the ratio between red and green representing a datapoint in statistics.

# Respiratory analysis

Two to five days prior to the experiment 60,000-85,000 hDaNs were seeded into a Seahorse cell plate coated with Matrigel. Respiration was measured in DMEM base medium (without phenol red; Sigma-Aldrich, #D5030 1L) supplemented with 4.5 mg/mL D-Glucose, 0.22 mg/mL pyruvate, and 2 mM glutamine with subsequent injections of (a) medium or5 µM ionomycin, (b) 0.8 µM Oligomycin, (c) 2.7 µM CCCP, and (d) 0.8 µM Rotenone with 4 µM Antimycin A. (a) was measured five times, (b-d) three times. Measurements were normalized to number of hDaNs seeded. Basal respiration after injection of (a) was calculated subtracting mean of measurements after (d) from mean after (a); spare respiratory capacity is the percentage of maximal respiration (mean after (c)-mean after(d)) and basal respiration (last basal measurement-mean (d).

# Calcium imaging

Calcium imaging – hDaNs

hDaNs were seeded on Matrigel in 35mm Ibidi dishes. Prior to imaging, hDaNs were first treated for 2 h with 2 µM Mitoxantrone and for imaging of cytosolic calcium then stained with FLUO-4 (Invitrogen, #F10471) following the manufacturer’s instructions. To chelate extracellular calcium, cells were incubated with 3µM EGTA for 10 min prior to stimulation with an acute injection of 5 µM Thapsigargin. Timelapses were taken using a Leica DMi8 epifluorescence microscope (20X objective, Leica LASX software) under controlled environment (37°C/5% CO2) in phenol red-free maturation medium (hDaN) + 2X FLUO-4 complete reagent. First the baseline reading was taken at 5 sec intervals for 3 min and then after stimulation with Thapsigargin the timelapse was taken in 5 sec intervals for 5 min. Timelapses were then processed, analyzed and measured with Fiji: the frames were used to draw a region of interest (ROI) around the Calcium firing neuronal cells and the single ROI´s signal intensity was measured. The resulting matrix of values, with every column corresponding to a single ROI per cell, and every row to a different frame, was processed and plotted using GraphPad Prism software. The calculations for F_MAX_/F_0_ were done by dividing the maximum FLUO-4 intensity value to the average FLUO-4 value of the baseline reading. The rate of FLUO-4 decline after reaching peak value was calculated using the formula – (F_MAX_/F_0_)/(485-t_MAX_), where 485 is the endtime and t_MAX_ is the time at which the F_MAX_ was attained.

Calcium imaging – SH-SY5Y

SH-SY5Ys were seeded on Collagen in 35mm Ibidi dishes. SH-SY5Y were transfected three consecutive days starting four days prior to imaging with non-targeting siRNA (Dharmacon; #D-001206-14-05) or RHOT1 siRNA (Dharmacon, #M-010365-01-0005) with transfection reagent (Dharmacon, #T-2001-01/T-2004-01) following the manufacturer’s instructions. Prior to imaging, SH-SY5Ys were first treated for 2 h with 2 µM and 10 µM Mitoxantrone, respectively, and for imaging of cytosolic calcium then stained with FLUO-4 (Invitrogen, #F10471) following the manufacturer’s instructions. To chelate extracellular calcium, cells were incubated with 3µM EGTA for 10 min prior to stimulation with an acute injection of 5 µM Thapsigargin. Timelapses were taken using a Leica DMi8 epifluorescence microscope (40󠇙X objective, Leica LASX software) under controlled environment (37°C/5% CO2) in maintenance medium in 5 sec intervals for 5 min using a Leica DMi8 Microscope (40X objective) with the LASX software. Timelapses were then processed, analyzed and measured with Fiji: the frames were segmented into single regions of interest (ROIs) corresponding to cells and the single ROI´s signal intensity measured. The resulting matrix of values, with every column corresponding to a single ROI or cell, and every row to a different frame, was processed through a MATLAB script. The script first normalized the matrix by subtracting the signal intensity corresponding to the background and then also subtracting the signal intensity corresponding to t_0_ of every ROI. Second the script partially corrected for desynchronization both intra- and inter-field of views by trying to align the curves following an arbitrary increase in signal intensity that would correspond to the beginning of a calcium transient. The curves generated from the output matrices of the MATLAB script were fitted according to the closest possible model using GraphPad Prism. The rise of the calcium signal was fitted to a linear increase, while the calcium buffering was fitted to a one phase exponential decay.

# Dopamine staining

Two days prior to staining, 50,000 hDaNs were seeded on PLO-Matrigel double-coated glass coverslips. hDaNs were treated for either 24 h with 5 µM L-DOPA or 0.5 h with 5 µM ionomycin before staining using STAINperfect (Immusmol, #SP-A-1000) following manufacturer’s instructions (secondary antibodies: Jackson ImmunoResearch, # 103-545-155; Thermo Fisher, A#11077, #A21245) with Hoechst stain for nuclei. For acquisition of 10 Z-stacks per differentiation with a slice interval of 1 µm, fixed exposure times for each channel were used. Images were processed and analyzed in Fiji. First, stacks were projected with average intensity and converted to 8-bit greyscale. For measuring area, mean grey value and integrated density MAP2 channel was used to define a ROI. MAP2 greyscale images were first converted into a binary image by applying thresholding after subtracting background with a rolling ball radius of 25 px, then the binary image was used to create a selection that was applied to greyscale images of all channels for measurement and calculation of CTF. Each image analyzed represents one data point in statistics.

# Measurement of MAO enzyme activity

For measurement of MAO enzyme activity, a crude mitochondrial preparation from hDaNs was performed. Neurons were washed with PBS before addition of mitochondria isolation buffer supplemented with protease and phosphatase inhibitors (Millipore Sigma, #11873580001 and Sigma-Aldrich, #4906837001). Detached cells were homogenized by pipetting and subsequent passes through different needle sizes (8x 20G/8x 27G/8x 30G). After centrifugation for 5 min at 1000 xg at 4°C, supernatant was saved and pellet was resuspended in mitochondria isolation buffer and further homogenized by eight passes through a 30G needle. After for 5 min at 1000 xg at 4°C, supernatant was pooled with previous one (pellet was discarded) and centrifuged for 15 min at 10,000 xg at 4°C. The supernatant (cytosolic fraction) was saved and the pellet (mitochondrial fraction) resuspended in MAO assay buffer (100 mM HEPES, 5% Glycerol, protease and phosphatase inhibitors). After measuring protein concentration using Bradford, 45-100 µg protein of mitochondrial fraction were used for measurement of MAO enzyme activity with MAO-Glo assay (Promega, #V1401); (Valley et al., 2006) following the manufacturer’s instructions using MAO-B reaction buffer. To assess specificity of the assay, inhibitors Clorgyline (abcam, #ab145646) and Deprenyl (abcam, #ab120604) were added to the assay in concentrations of 0.001/0.01/0.1/1/10 µM. As negative control, the enzyme activity was measured without enzyme. Chemiluminescence was measured after incubation with the detection reagent for 30 min at room temperature using a FLUOstar OMEGA (BMG Labtech). Obtained values were background corrected using the negative control and for amount of protein used. As a control experiment, citrate synthase activity was measured (Shepherd and Garland, 1969). 10 µg protein (isolated mitochondria) was mixed with assay buffer (100 mM Tris pH 8.0, 0.1% TritonX-100), 10 µM Acetyl-CoA and 200 µM DTNB (freshly dissolved in water). Reaction was started by addition of 20 µM Oxaloacetate (freshly dissolved in water) or assay buffer as reference and measured at room temperature for 10 min with a 30 sec interval at 412 nm using a SpectraMax M2e plate reader (Molecular Devices). Each value was normalized to t=0 prior to calculation of the slope, which was normalized to amount of protein used for the assay.

# Transcriptomics

RNA quality from three independent hDaN differentiations was assessed with an Agilent 2100 Bioanalyzer and the Agilent RNA 6000 Nano kit (Agilent). Samples with very high RNA integrity number were selected for library construction. For polyA enrichment, total RNA was subjected to polyA enrichment and cDNA libraries were constructed using the resulting mRNA and the NEBNext Ultra II Directional RNA Library Prep Kit (New England BioLabs). Libraries were sequenced on a NextSeq500 (Illumina) with a depth of >25 million reads each. Library preparation and sequencing procedures were performed by the same individual and a design aimed to minimize technical batch effects was chosen. Read quality of RNA-seq data in fastq files was assessed using QoRTs to identify sequencing cycles with low average quality, adaptor contamination, or repetitive sequences from PCR amplification. Reads were aligned using STAR allowing gapped alignments to account for splicing against a custom-built genome composed of the Ensembl Homo Sapiens GRCh37 and Alignment quality was analyzed using samtools and visually inspected in the Integrative Genome Viewer. Normalized read counts for all genes were obtained using Subread and edgeR. EdgeR uses an exact test for the negative binomial distribution, which has strong parallels with Fisher's exact test, to compute exact *p* values that can be used to assess differential expression. Transcripts covered with less than 1 count-per-million in at least 1 sample were excluded from the analysis for determining differential expression in each of the pair-wise comparisons between experimental groups. For depicting the results in a volcano plot, differentially regulated transcripts were added to a GraphPad Prism file and -log(FDR) and log(FC) values were used as y and x values, respectively. As threshold (indicated in graph by line), a -log(FDR) of 1.7 and log(FC) of ±1 were chosen; values above both thresholds were considered as up- and downregulated and labelled with red and green dots, respectively. Targeted pathway analysis using Qiagen software Ingenuity was performed on the pathways dopamine receptor signalling and synaptic long term potentiation. For Gorilla pathway analysis (Eden et al., 2007, Eden et al., 2009) all differentially regulated genes with p < 0.5 were chosen as target dataset and all detected genes as background dataset. The generation of *p* values by the Gorilla pathway algorithm is described in Eden et al., 2009. The top five results in the categories function, process and component were used for the figure. For displaying of mitochondria related and synapse related genes, we plotted the selected genes on the y axis and their corresponding log(FC) on the x axis. *P* values indicate difference between isogenic control and Miro1 R272Q. We provide the list of transcripts (CTRL versus Miro1 R272Q) ranked by LogFC and significance, which was used to run the Gorilla pathway analysis in Supplementary Table 1.

# References

EDEN, E., LIPSON, D., YOGEV, S. & YAKHINI, Z. 2007. Discovering motifs in ranked lists of DNA sequences. *PLoS Comput Biol,* 3**,** e39.

EDEN, E., NAVON, R., STEINFELD, I., LIPSON, D. & YAKHINI, Z. 2009. GOrilla: a tool for discovery and visualization of enriched GO terms in ranked gene lists. *BMC Bioinformatics,* 10**,** 48.

HERNANDEZ, G., THORNTON, C., STOTLAND, A., LUI, D., SIN, J., RAMIL, J., MAGEE, N., ANDRES, A., QUARATO, G., CARREIRA, R. S., SAYEN, M. R., WOLKOWICZ, R. & GOTTLIEB, R. A. 2013. MitoTimer: a novel tool for monitoring mitochondrial turnover. *Autophagy,* 9**,** 1852-61.

KOCSIS, E., TRUS, B. L., STEER, C. J., BISHER, M. E. & STEVEN, A. C. 1991. Image averaging of flexible fibrous macromolecules: The clathrin triskelion has an elastic proximal segment. *Journal of Structural Biology,* 107**,** 6-14.

SHEPHERD, D. & GARLAND, P. B. 1969. The kinetic properties of citrate synthase from rat liver mitochondria. *Biochemical Journal,* 114**,** 597-610.

TINEVEZ, J. Y., PERRY, N., SCHINDELIN, J., HOOPES, G. M., REYNOLDS, G. D., LAPLANTINE, E., BEDNAREK, S. Y., SHORTE, S. L. & ELICEIRI, K. W. 2017. TrackMate: An open and extensible platform for single-particle tracking. *Methods,* 115**,** 80-90.

VALLEY, M. P., ZHOU, W., HAWKINS, E. M., SHULTZ, J., CALI, J. J., WORZELLA, T., BERNAD, L., GOOD, T., GOOD, D., RISS, T. L., KLAUBERT, D. H. & WOOD, K. V. 2006. A bioluminescent assay for monoamine oxidase activity. *Anal Biochem,* 359**,** 238-46.
